# Immunohistochemical Character of Hepatic Angiomyolipoma: For Its Management

**DOI:** 10.1155/2013/298143

**Published:** 2013-06-20

**Authors:** Yuka Kobayashi, Kenya Kamimura, Minoru Nomoto, Soichi Sugitani, Yutaka Aoyagi

**Affiliations:** ^1^Division of Gastroenterology, Tachikawa General Hospital, Kandamachi 3-2-11, Nagaoka, Niigata 940-8621, Japan; ^2^Division of Gastroenterology and Hepatology, Graduate School of Medical and Dental Sciences, Niigata University, Asahimachi 1-757, Chuo-ku, Niigata, Niigata 951-8122, Japan

## Abstract

Hepatic angiomyolipoma (AML) is notoriously difficult to diagnose without an invasive surgery even with the recent development of the various imaging modalities. Additionally, recent reports showed its malignant behavior after the surgery; it is important to diagnose the character of each tumor including the possible malignant potential and determine the postoperative management for each case. For this purpose, we have reviewed reports and focused on the immunohistochemical staining with p53 and ki67 of the tumors showing the representative case of 60-year-old female. The imaging study of her tumor showed the character similar to the hepatocellular carcinoma, and she underwent the hepatectomy. The resected tumor stained positive for HMB-45 that is a marker of the AML, and 30–50% of the tumor cells were positively stained with Ki67 that is a mitotic marker. Also, the atypical epithelioid cells displayed p53 immunoreactivity. These results suggest the malignant potential of our tumor based on the previous reports; therefore the careful followup for this case is necessary for a long period whether it shows metastasis, sizing up, and so forth.

## 1. Introduction

Angiomyolipoma of the liver is rare and has been considered a benign tumor since Ishak [1] first described the condition in 1976. The tumor has three cellular components: fat cells, smooth muscle cells, and blood vessels. However, the proportion of these three components varies considerably from case to case and from area to area within the same tumor. The classification of malignant liver tumors is often very difficult due to the phenotypic variability of the fatty portion [2–4]. In histological preparations, the smooth muscle cell element of the tumor exhibits variable morphological features with occasional atypical cells. These tumors are frequently misdiagnosed as malignant neoplasms. It is well known that an invasive growth pattern is one of the most important histological features differentiating malignant from benign tumors. Although the majority of hepatic AML are clinically benign, invasive growth features are frequently found in hepatic AML [5]. However, malignant hepatic AML is extremely rare; distant metastasis and tumor recurrence are rarely reported. Dalle et al. reported a 70-year-old patient with hepatic AML that showed prominent vascular invasion histologically, and the patient died of recurrent disease with multiple liver and peritoneal metastases [6]. Certain hepatic AML characteristics may be associated with malignant potential. These include tumor size, portal vein thrombus, marked cell proliferation, and p53 immunoreactivity, p53 mutations [7, 8]. Based on these facts, although the imaging studies have been developed with the new modalities, the histological diagnosis and the surgical treatment still play a key role to determine the management strategy for each case. Therefore, in this paper, showing the representative case, we reviewed the literature and discussed the importance of the immunohistochemical staining with p53 and ki67 for the management of this tumor.

## 2. Diagnosis of Hepatic AML

Among the various benign tumors, angiomyolipoma (AML) is a well-known renal tumor associated with the tuberous sclerosis complex. However, such tumors have been reported to occur in various extrarenal sites, including the liver, uterus, and retroperitoneum. Hepatic AML is a rare benign tumor of mixed mesenchymal origin, which Ishak [1] firstly described in 1976. The disease is asymptomatic in 60% of patients; abdominal pain is the most common symptom [9, 10]. Due to the variable amount of adipose tissue in each tumor, it is sometimes difficult to distinguish between benign and malignant tumors by imaging studies. Without the information provided by a surgical examination, it is possible to misdiagnose hepatic AML as a lipoma, hepatocellular carcinoma, sarcoma, or metastasis [11, 12]. While reviewing these reports, we have summarized the typical imaging findings for hepatic AML as follows: (1) ultrasonography (US) evidence of a heterogeneously hyperechoic mass, (2) plain computed tomography (CT) sign of a heterogeneously area of low density, and (3) magnetic resonance imaging (MRI) showing various images depending on the component of tumor tissue. High intensity on the T2-weighted image and high or low intensity on the T1-weighted image are observed based on the amount of adipose tissue contained. The adipose tissue is determined by the low-intensity area revealed in the out-of-phase and fat suppression images [13–16], (4) tumor staining and hypervascularity as revealed by angiography [13, 14]. Although a combination of US, abdominal CT, MRI, and angiography increases diagnostic accuracy, only 25%–52% of preoperative diagnoses are correct [15, 16]. Therefore, the definitive diagnosis is only possible postoperatively due to the need for histological verification. Even then, hepatic AML is often mistaken as hepatocellular carcinoma since it is rare. Identification of smooth muscle cells, blood vessels, and adipose tissue with a positive immunohistochemical reaction for HMB-45 is the final evidence for an angiomyolipoma [17–19]. The majority of hepatic AMLs invade the surrounding tissue. In this case, the intraoperative view revealed invasive growth, with hepatic cord hepatocyte replacement and extension into the portal area and/or around hepatic veins [5].

The biological behavior of hepatic AML requires further investigation. Because hepatic AML has been considered as benign tumor, most clinicians advocated conservative treatment. In 2000, Dalle et al. reported the first case of malignant hepatic AML [6], in a 70-year-old patient with hepatic AML that showed prominent vascular invasion upon histological examination. The patient died of recurrent disease with multiple liver and peritoneal metastases 7 months after the surgery. Since then, several authors have reported that hepatic AML is likely to metastasize, enlarge, and recur. Therefore, it is not prudent to treat hepatic AML as a simple benign tumor. At the very least, the physicians must be aware of the potential for malignant transformation.

We reviewed the literature [20, 21] and here summarized the similarities and differences between classic and malignant hepatic AML (Table 1). Both classic and malignant hepatic AML possess three basic components that can be visualized through histological investigation: blood vessels, fat cells, and epithelioid-spindle cells. Both types of hepatic AML express smooth muscle and melanocytic markers, such as *α*-SMA and HMB-45. In the nine cases that have been published, all of which clearly involved metastasis and/or recurrence, the malignant hepatic AML was >8 cm in diameter. Some cases involved portal vein thrombus and necrosis. A recent study by Maklouf et al. showed that CD117 staining was positive in all cases of benign renal and hepatic AML [22]. Nguyen et al. reported a loss of CD117 expression as a sign of hepatic AML malignancy [20]. Deng et al. reported that the central hepatic AML lesion could be identified as atypical epithelioid components with pleomorphic and frequent mitotic figures, p53 immunoreactivity, and p53 mutations at exon 7. High levels of p53 expression are often associated with malignancy, which means that positive p53 staining may signal impending metastasis [21]. Mizuguchi et al. stain for HMB-45 and Ki67, a mitotic marker, to identify hepatic AML malignancy [23]. Although all these findings support the existence of malignant hepatic AML, the reports leave many questions unanswered. Therefore, we are focusing on the immunohistochemical character of hepatic AML to predict its malignant potential for the decision of the management of the tumor.

## 3. Representative Case

Our representative case was a 60-year-old woman. She had no history of malignant disease, and the blood exams were negative for tumor markers and viral markers.

### 3.1. Imaging Studies

**Figure 1:**
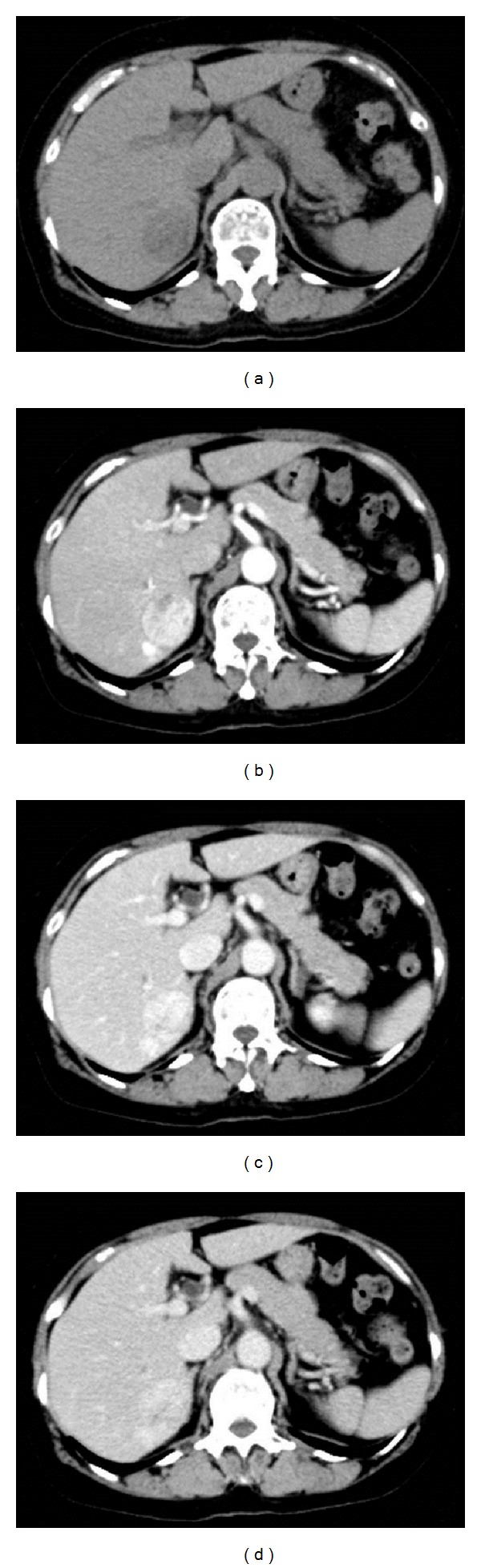
Dynamic contrast enhancement computed tomography (CT). Plain CT revealed a low-density tumor in segment 6 (a). Tumor hypervascularity in the arterial phase (b). Hypervascular lesions remained in the portal phase (c) and late phase (d).

**Figure 2:**
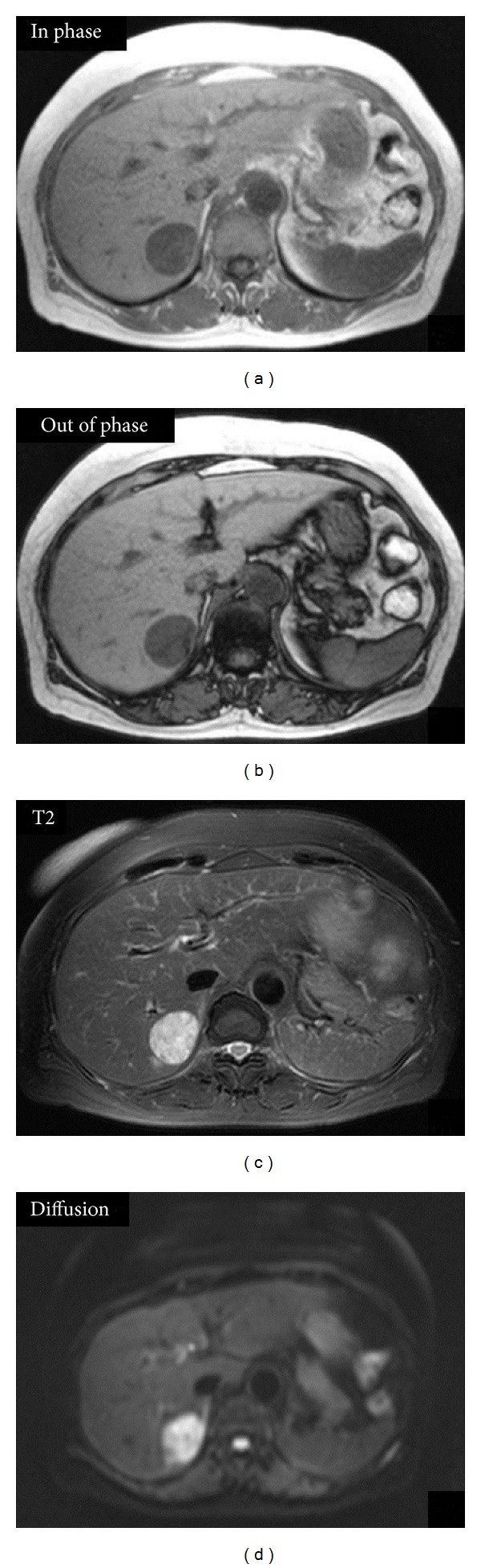
Magnetic resonance imaging. The tumor appeared as a low-intensity area on T1-weighted in-phase (a) and out-of-phase images (b). On T2-weighted with fat saturation and diffusion-weighted images, the tumor appeared as an area of high intensity ((c), (d)).

**Figure 3:**
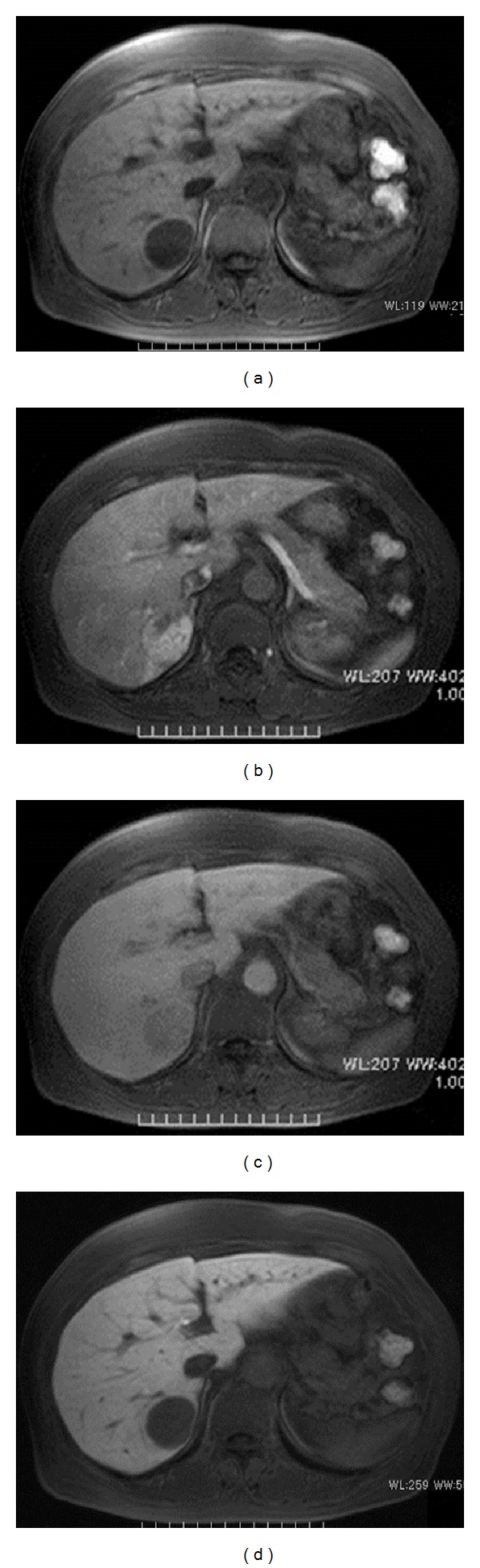
Dynamic contrast enhancement MRI with a hepatocyte-specific contrast agent (gadolinium-ethoxybenzyl-diethylenetriamine pentaacetic acid). Plain T1-weighted MRI revealed a low-intensity tumor (a), arterial phase hypervascularity (b), and a defective area visible in the late phase (c) and in the hepatic cellular phase (d).

The abdominal plain CT revealed a low-density area (Figure 1(a)) in segment 6 of the liver, and a dynamic study revealed a remarkably hypervascular tumor, which stained in the early phase (Figure 1(b)) and showed a vaguely defective area with minimal high-density staining in the late phase (Figures 1(c) and 1(d)). Abdominal ultrasonography was not performed. T1-weighted magnetic resonance imaging (MRI) depicted the tumor as an area of low signal intensity on in-phase (Figure 2(a)) and as a low-intensity lesion on out-of-phase (Figure 2(b)). Interestingly, the relative signal loss in out-of-phase is detected compared to the in-phase, probably due to the presence of the adipose tissue in the tumor. In diffusion-weighted images, the tumor was identified as an area of high signal intensity (Figures 2(c) and 2(d)). Dynamic contrast enhancement MRI with a hepatocyte-specific contrast agent (gadolinium-ethoxybenzyl-diethylenetriamine pentaacetic acid) revealed hypervascularity and early enhancement of the tumor. Hepatic cell phase images revealed defective areas within the tumor (Figures 3(a)–3(d)). On the basis of these imaging studies and the tumor location, we suspected malignancy associated with a hepatocellular carcinoma; therefore, she underwent the partial hepatectomy.

### 3.2. Pathology

**Figure 4:**
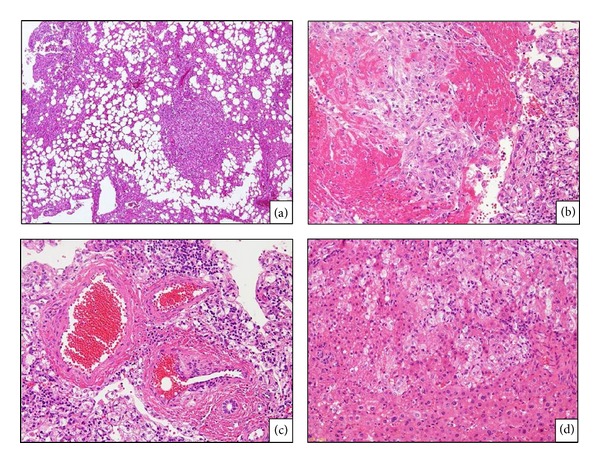
Histological findings (HE staining): the tumor included mature fat, blood vessels, and epithelioid-spindle cells (×120) (a). A part of the tumor showed predominance of spindle cells and blood vessel (×120) (b) (×300) (c). The tumor showed invasive growth pattern (×300) (d).

**Figure 5:**
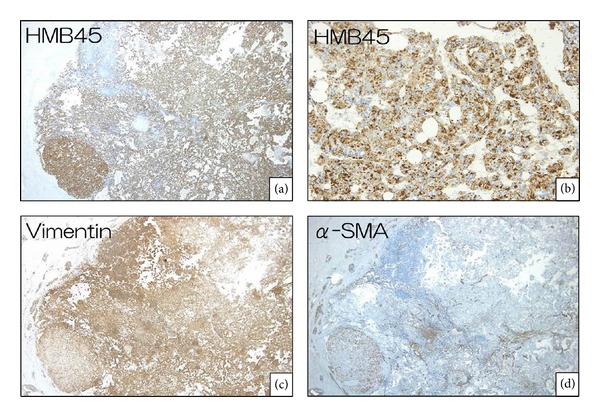
Immunopathological characteristics: tumor cells were positive for HMB-45 staining (×120) (a), (×1200) (b), for vimentin staining (×120) (c), and for *α*-SMA staining (×120) (d).

**Figure 6:**
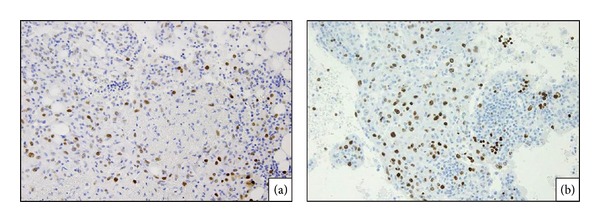
Immunopathological characteristics: approximately 5% of atypical epithelioid cells were positive for p53 (×1200) (a), and more than 30% of epithelioid cells were immunoreactive for Ki67 (×1200) (b).

A partial hepatectomy of segment 6 was performed. Upon macroscopic examination, the surgical specimen appeared as a solid whitish mass consisting of a single nodule free of necrosis. There were no signs of chronic inflammation or fibrosis in the surrounding liver tissue. The three defining characteristics of AML were identified, including mature fat, blood vessels, and epithelioid-spindle cells (Figures 4(a)–4(c)). The round or polygonal tumor cells were arranged in a sheet, with sign of tumor invasion at the tumor-nontumor interface (Figure 4(d)). Tumor cells had replaced hepatocytes within the liver cell cords along the hepatic sinusoids. In addition, small isolated clusters of tumor cells were occasionally found proximal to the main tumor mass, suggesting tumor sprouting. The tumor was positive for HMB-45 (Figures 5(a) and 5(b)), a marker of AML, vimentin, and *α*-SMA (Figures 5(c) and 5(d)). Fat cells and blood vessels were observed as well, and interestingly p53-positive cells were scattered diffusely throughout the tumor (Figure 6(a)), and 30%–50% of cells in the solid region of the tumor were positive for Ki67 (Figure 6(b)). Based on the literature review described above, we diagnosed that this hepatic AML might have a malignant potential, therefore, this patient is being followed carefully at our hospital, and, to date, there has been no evidence of postoperative recurrence or metastasis.

## 4. Conclusion

Hepatic AML usually follows a benign clinical course. However, the tumor malignancy cannot be ignored. Therefore, we recommend that excision or fine-needle biopsy be accompanied by subsequent histological staining for markers such as Ki67 and p53. This information will be helpful for the management of the tumor.

## Figures and Tables

**Table 1:** Characteristics for malignant AML.

	Classic AML components	Atypical AML components
Tumor size [7]		>5 cm
Pathological		
Cell atypical	−	+
Invasion in hepatic parenchyma	±	+
Portal venous tumor thrombus [7]	−	+
Necrosis [20]	−	+
Immunohistochemical		
HMB-45	+	+
*α*-SMA	+	+
Ki67 [23]	<5%	>30%
p53 [21]	−	>10%
CD117 [20]	+	−
